# Study on Degradation of Sulfamethoxazole in Water by Activated Persulfate of Molybdenite Supported on Biochar

**DOI:** 10.3390/molecules31020211

**Published:** 2026-01-07

**Authors:** Xuemei Li, Jian Wang, Xinglin Chen, Shengnan Li, Hai Lu

**Affiliations:** 1 College of Visual Arts, Changchun Sci-Tech University, Changchun 130600, China; 100464@cstu.edu.cn; 2 Basic Research Department, Changchun Sci-Tech University, Changchun 130600, China; 100495@cstu.edu.cn; 3 Key Laboratory of Songliao Aquatic Environment, Ministry of Education, Jilin Jianzhu University, Changchun 130118, China; chenxinglin@student.jlju.edu.cn (X.C.); lishengnan@neau.edu.cn (S.L.)

**Keywords:** biochar, molybdenite, peroxymonosulfate, sulfamethoxazole, degradation efficiency, degradation mechanism

## Abstract

In this study, the advanced oxidation system of peroxymonosulfate (PMS) was activated by molybdenite supported on biochar (Molybdenite@BC), and the degradation efficiency, influencing factors and degradation mechanism of sulfamethoxazole (SMX) were explored through experiments. Molybdenite@BC, a composite material used in the study, was prepared by pyrolysis at high temperature. The optimum pyrolysis temperature was 700 °C, and the mass ratio of molybdenite to biochar (BC) was 1:3. By changing dosage of Molybdenite@BC, pH value, initial concentration of PMS, and the types and concentration of inorganic anions, the effects of various factors on SMX degradation were systematically studied. The optimum reaction conditions of the Molybdenite@BC/PMS process were as follows: Molybdenite@BC dosage was 100 mg/L, PMS concentration was 0.2 mM, pH value was 6.9 ± 0.2, and initial SMX concentration was 6 mg/L. Under these conditions, the degradation rate of SMX was 97.87% after 60 min and 99.06% after 120 min. The material characterization analysis showed that Molybdenite@BC had a porous structure and rich active sites, which was beneficial to the degradation of pollutants. After the composite material was used, the peaks of MoO_2_ and MoS_2_ became weaker, which indicated that there was some loss of molybdenum from the material structure. Electron paramagnetic resonance (EPR) and radical quenching experiments revealed that Molybdenite@BC effectively catalyzed PMS to generate various reactive oxygen radicals and non-free radicals, including singlet oxygen (^1^O_2_), hydroxyl radical (^•^OH), sulfate radical (SO_4_^•−^) and superoxide radical (^•^O_2_^−^). ^1^O_2_ played a leading role in the degradation of SMX, while ^•^OH and SO_4_^•−^ had little influence. The intermediate products of the degradation of SMX in Molybdenite@BC/PMS system were analyzed by liquid chromatography–tandem mass spectrometry (LC–MS). The results showed that there were nine main intermediate products in the process of degradation, and the overall toxicity tended to decrease during the degradation of SMX. The degradation path analysis showed that with the gradual ring opening and bond breaking of SMX, small molecular compounds were generated, which were finally mineralized into H_2_O, CO_2_, CO_3_^2−^, H_2_SO_4_ and other substances. The research results confirmed that the Molybdenite@BC/PMS process provided a feasible new method for the degradation of SMX in water.

## 1. Introduction

Sulfonamide antibiotics, as an important class of antibacterial drugs, have been widely used in medical treatment and animal husbandry for a long time because of their significant inhibitory effect on both gram-positive bacteria and gram-negative bacteria. However, its ubiquity in the water environment and its potential ecological risks have attracted widespread attention [1,2,3]. Sulfamethoxazole (SMX) belongs to sulfonamides, and it is one of the most widely used antibiotics in the world [4,5,6,7]. The molecular formula of SMX is C_10_H_11_N_3_O_3_S, which is a white crystalline powder and belongs to organic compounds. This substance is odorless, slightly bitter in taste, and insoluble in water, but easily soluble in dilute hydrochloric acid, sodium hydroxide reagent or ammonia reagent, and also soluble in methanol and ethanol. Due to extensive use, SMX was frequently detected in the environment and was confirmed as a persistent pollutant.

SMX is a typical sulfonamide antibiotic. At present, the removal methods of these antibiotics in water environment mainly include biological method, physical adsorption method, chemical method and advanced oxidation processes (AOPs).

(1) Biological method. Biological method is an effective method to remove organic pollutants by biochemical action of microorganisms or plants, which mainly includes aerobic treatment, anaerobic treatment and constructed wetland treatment [8]. The research results of Carballa et al. [9,10] showed that the traditional activated sludge process could remove about 40~65% of SMX. Batt et al. [11] found that the removal effect of trimethoprim by conventional activated sludge was poor, but the removal rate could increase to 50% after the nitrification activated sludge was introduced.

(2) Physical adsorption method. Physical adsorption is regarded as an extremely effective way to remove toxic chemical pollutants in water and air [12]. Common adsorption materials include activated carbon, diatomite, zeolite and synthetic resin. The effect of adsorption technology is affected by pH, contact time, pollutant concentration and ionic strength. In the study of adsorption and removal of sulfonamides, Tonucci et al. [13] compared powdered activated carbon prepared by different steam activation, and found that the adsorption performance of the material was related to the pore structure. The magnetic ion exchange resin developed by Wang et al. [14] showed excellent adsorption capacity for SMX (the maximum adsorption capacity could reach 789.32 μg/mL), and its adsorption mechanism mainly involved anion exchange and hydrogen bonding. Kolpak et al. [15] found that the increase in oxygen-containing functional groups on the surface of carbon nanotubes after oxidation would reduce the hydrophobicity of the materials, weaken their affinity with SMX, and eventually led to the decline of adsorption efficiency. Although adsorption technology had good removal effect on sulfonamides, there are also problems, such as difficult regeneration of adsorbents and high treatment cost [16].

(3) Chemical oxidation method. Chemical oxidation is a treatment technology that destroys the molecular structure of antibiotics by strong oxidants and transforms them into low-toxic or non-toxic small molecular substances [17,18]. Commonly used oxidants include ozone, chlorine-containing compounds, potassium permanganate and ferrate, etc. Studies have shown that [17], ozone showed strong reactivity to organic compounds containing unsaturated bonds (such as olefins and aromatic compounds) and deprotonated amino functional groups. The traditional chemical oxidation method has two defects: first, a large amount of oxidant is often needed to achieve the expected treatment effect; secondly, the oxidation process may produce higher-risk intermediate products. For example, research showed that [18], although chlorine dioxide and free chlorine can efficiently degrade sulfonamides and macrolide antibiotics in sewage, chlorite with strong toxicity will be produced during the oxidation of sulfonamides.

(4) Advanced oxidation method. In the field of environmental science and engineering, advanced oxidation is generally regarded as a promising and forward-looking technical means to treat organic wastewater [19]. The core mechanism of advanced oxidation method is to achieve efficient degradation of organic pollutants by producing strongly oxidizing species, such as hydroxyl radical (^•^OH). Compared with traditional physical adsorption or biological methods, AOPs have high degradation efficiency and complete mineralization. Especially for refractory organic matter, AOPs can mineralize it into CO_2_ and H_2_O.

In the study of antibiotic degradation, AOPs have been widely studied because they can produce highly reactive free radicals to oxidize and degrade the target pollutants, and have a remarkable effect in treating antibiotic wastewater [20,21]. In recent years, the AOPs based on persulfate activation to produce SO_4_^•−^ with higher selectivity and longer half-life have made important progress. This technology has obvious advantages, such as fast reaction rate, low selectivity, and being able to deal with refractory organic pollutants. At present, the commonly used persulfate activation technologies mainly include ultraviolet light activation, transition metal activation and carbon material activation.

(1) Ultraviolet light (UV) activation. Generally, when ultraviolet light is used to activate peroxydisulfate (PDS), light with a wavelength of 254 nm is widely used. This is because PDS can exhibit a maximum quantum yield of 1.4 under the irradiation of ultraviolet light (UV_254_) of about 254 nm. In contrast, when UV_254_ is used to activate peroxymonosulfate (PMS), the quantum yield is only 0.52 [22]. Under the irradiation of ultraviolet light, the O–O bond in PDS and PMS breaks, which leads to a series of complex photochemical reactions, and finally produces SO_4_^•−^ and ^•^OH. The reaction process is shown in Formulas (1) and (2).

(1) $${{\mathrm{UV}:\;S}_{2}O_{8}}^{2-}\;\to \;2{{\mathrm{SO}}_{4}}^{•-}$$

(2) UV:HSO5− → SO4•−+OH•

(2) Transition metal activation. The research results show that [23,24], Ag^+^ has the most outstanding activation effect on PDS; Co^2+^ has the most significant activation effect on PMS. The activation mechanism is shown in Formulas (3) and (4).

(3) $${S_{2}O_{8}}^{2-}+M^{n}\;\to \;{{\mathrm{SO}}_{4}}^{•-}+{{\mathrm{SO}}_{4}}^{2-}+M^{n+1}$$

(4) $${HSO}_{5}^{-}+M^{n}\;\to \;{{\mathrm{SO}}_{4}}^{•-}+{OH}^{-}+M^{n+1}$$

Nowadays, a lot of research has been devoted to synthesizing heterogeneous materials to activate PDS and PMS. As an environment-friendly catalyst, natural mineral materials show important application value in the field of heterogeneous activation of persulfate. Wang et al. [25] made a α–MnO_2_ material to activate PMS for degrading phenol. The results showed that phenol could be completely degraded after 60 min of reaction. Virkutyte et al. [26] comparatively studied the catalytic effect of Fe_2_O_3_–montmorillonite on PMS, H_2_O_2_ and peracetic acid. The results show that the PMS system showed excellent mineralization performance and the mineralization rate of dichlorophenol (DCP) could reach 85% in 2.5 h; however, in the system of H_2_O_2_ and peracetic acid, the mineralization rate reached only 70% and 50%, respectively, after a 3.5 h reaction.

(3) Activation of carbon materials. Carbon-based catalysts show remarkable versatility in the field of environmental remediation, especially materials, such as activated carbon and graphene, that have dual functions in wastewater treatment [27]. The mechanism is shown in Formulas (5) and (6).

(5) $${S_{2}O_{8}}^{2-}+e^{-}\;\to \;{{\mathrm{SO}}_{4}}^{•-}+{{\mathrm{SO}}_{4}}^{2-}$$

(6) $${HSO}_{5}^{-}+e^{-}\;\to \;{{\mathrm{SO}}_{4}}^{•-}+{OH}^{-}$$

Among many carbon materials, except graphene and carbon nanotubes, BC has been widely studied in the environmental field because of its low cost. Fang et al. [28] studied the degradation efficiency of polychlorinated biphenyls (PCBs) by activated PDS with BC, and confirmed that biochar could effectively activate PMS to generate SO_4_^•−^, and the removal rate could reach 70~100% within 30 min. In order to optimize the performance of carbon-based catalysts, researchers often modify them by doping N, O, S, P and other elements or metal oxides, thus synthesizing new materials with better performance [29].

Natural mineral–biochar composite material is a kind of composite material prepared by co-pyrolysis of natural minerals and biochar. Molybdenite is molybdenum disulfide (MoS_2_) and the main source of molybdenum. Molybdenite, including hexagonal and triangular crystal systems and other different types, has a typical layered structure, low hardness, usually in scaly or fine-grained form, with a lead-gray appearance and strong metallic luster. MoS_2_ has a good activation effect on PMS and is widely used in AOPs [30].

In view of the good performance of molybdenite in AOPs, in this study, natural molybdenite was used as the main component of a composite catalytic material, and waste corn stalk was used as biomass to prepare a new PMS catalyst, namely, biochar-supported molybdenite (Molybdenite@BC). It was a composite material prepared by dispersing Molybdenite on BC. On this basis, in the Molybdenite@BC/PMS process, the degradation efficiency of the system within 2 h was investigated and the mechanism was analyzed by exploring the initial concentration of SMX, pH value, initial concentration of PMS, dosage of composite materials, and the effects of three common inorganic anions (HCO_3_^−^, Cl^−^ and SO_4_^2−^) and humic acid (HA) in water on the reaction.

The Molybdenite@BC/PMS system constructed in this study is not only technically related to the existing research, but also shows remarkable innovation. Previous studies have focused on the degradation of SMX by activating PMS with different catalysts (hematite supported cobalt, straw-based biochar, iron-based redox medium, etc.). Although high degradation efficiency has been achieved, there are limitations, such as complex catalyst preparation, narrow pH application range, and the dependent free radical path is easily disturbed by water matrix [16]. The highlight of this study is that Molybdenite@BC composite is constructed with corn straw biochar as the carrier, which not only solves the problem of MoS_2_ agglomeration by using the porous structure of BC, but also realizes the stable load of molybdenum ore through Mo=O bond. The system takes ^1^O_2_ as the leading non-radical path, and its anti-interference ability is stronger. Meanwhile, the optimum preparation conditions were determined: molybdenite and BC pyrolyzed at 700 °C with a mass ratio of 1:3. Compared with the unoptimized pure MoS_2_ system [30], the degradation efficiency of SMX was improved by about 20%, and the catalyst cost was lower. This composite catalyst can be prepared from agricultural wastes, which has more practical application value.

## 2. Materials and Methods

### 2.1. Materials and Reagents

The reagents used in the experiment were as follows. Sulfamethoxazole (C_10_H_11_N_3_O_3_S, 99%), formic acid (HCOOH, ≥99%), L–histidine (C_6_H_9_N_3_O_2_, 99%), and P–benzoquinone (C_6_H_4_O_2_, 99%) were all produced by Shanghai Macklin Biochemical Co., Ltd. (Shanghai, China). Sodium hydroxide (NaOH, AR), sodium chloride (NaCl, AR), sulphuric acid (H_2_SO_4_, AR), tertiary butyl alcohol (C_4_H_10_O, AR), sodium bicarbonate (NaHCO_3_, GR), absolute ethyl alcohol (C_2_H_6_O, AR), muriatic acid (HCl, AR), and ethanol (C_2_H_6_O, AR) were all produced by National Medicine Group Chemical Reagent Co., Ltd. (Nanjing, China). Potassium peroxymonosulfate (KHSO_5_, AR), methyl alcohol (CH_3_OH, ≥99.9%) were produced by Sigma Aldridge Gmbh (Schnelldorf, Germany).

In the experiment, all solutions were prepared by an ultra-pure water machine (Molelement elemental type 1820a water system, Molecular scientific instrument limited company, Shanghai, China).

### 2.2. Preparation of Activated Composites

(1) The corn stalks were washed by tap water until the water was clear, washed by pure water three times and then dried. The straw was crushed by a crusher and screened by a 200-mesh sieve (74 μm). Then, the straw was placed in a vacuum oven (Lichen Scientific Instrument (Zhejiang) Co., Ltd., Shanghai, China) for preliminary pyrolysis at 200 °C for 120 min. After the sample was cooled to room temperature, it was put into a self-sealed bag for later use.

(2) Molybdenite was made of high-purity molybdenite crushed stone (Mo content was 59.94%, S content was 40.06%) produced in Jiangxi Province, China, which was washed with acid three times, washed with water three times, dried and ground, and screened with a 200-mesh screen for later use.

(3) In order to determine the best preparation conditions of molybdenite loaded with BC, the composite ratios of molybdenite and BC were set to be 1:0.5, 1:1, 1:1.5, 1:2 and 1:3, respectively, to form five materials with different composite ratios. Molybdenite powder and corn stalk BC powder was put in a beaker, and 80 mL pure water was added. After ultrasonic treatment for 10 min and stirring with a magnetic stirrer for 4.5 h, a mixed sample was obtained. The above samples were dried in an oven at 80 °C for 12 h, taken out and ground, and screened by a 200-mesh sieve for pyrolysis.

(4) Five pyrolysis temperatures were set in the experiment, which were 400 °C, 500 °C, 600 °C, 700 °C and 800 °C, respectively. The ground and sieved samples were put into a crucible for flattening and compaction, and put in a muffle furnace. When the required pyrolysis temperature was reached, the muffle furnace was closed and let it cool naturally. The ground and sieved samples were flattened and compacted in a crucible, and then put into a muffle furnace. After the oven door was closed, timing started (4 h) when the temperature reached the set temperature. The power supply of the muffle furnace was turned off after 4 h, then the sample was taken out after natural cooling, and marked for later use.

### 2.3. Experimental Process

The degradation experiment of SMX was carried out in a 250 mL conical flask with stopper. At room temperature, the composite material and PMS were added into the reaction system, then placed in a constant temperature oscillator at 25 °C, and operated at a speed of 165 r/min for reaction. After reacting for 0, 5, 10, 15, 30, 45, 60, 90 and 120 min, respectively, 1 mL of reaction solution was taken out and put into a liquid-phase vial filled with 0.2 mL of absolute ethanol. Then it was filtered with 0.22 μm organic phase filter membrane (PTFE membrane polypropylene housing, Merck KGaA, Darmstadt, Germany) and used for detection. Two groups of parallel samples were set up in each experiment. The concentration of SMX was 6 mg/L, the concentration of PMS was 0.15 mM, and the mass of composite material was 100 mg/L. The volume of sample in each 250 mL conical flask was 100 mL, and the pH of sample was adjusted by using 30% HCl solution and NaOH solution.

### 2.4. Analysis and Detection Methods

The residual concentration of SMX was analyzed by high performance liquid chromatography (HPLC, Agilent 1260 Infinity II, Santa Clara, CA, USA). The column Agilent Zorbax SB–C18 (4.6 × 150 mm, 5 μm) was used for separation. The column temperature was kept at 30 ± 1 °C, and the mobile phase was mixed with 0.1% formic acid water and methanol at a volume ratio of 7:3, and the flow rate was set at 0.8 mL/min. The sample volume was 50 μL and the detection wavelength was 286 nm. The peak time was about 4.3 min.

A TESCAN MIRA LMS scanning electron microscope (Quattro S, Brno, Czech Republic) was used to observe the morphology and energy spectrum of the samples. The total specific surface area, total pore volume and pore diameter of materials were measured by an automatic specific surface area and porosity analyzer (ASAP2460, Norcross, GA, USA). The functional groups on the surface of BC were tested by a Thermo Scientific Nicolet iS20 instrument (iS20, Waltham, MA, USA). The crystal structure before and after the reaction was analyzed by Rigaku SmartLab SE (SmartLab SE, Tokyo, Japan). In the test, the copper target was selected as the test target, the scanning angle ranged from 5 to 90°, and the scanning speed was 5° min^−1^. Thermo Scientific K–Alpha X-ray photoelectron spectroscopy (XPS) instrument (K–alpha, Waltham, MA, USA) was used for XPS characterization analysis to distinguish the metal valence state of Mo on the material surface.

Ultra performance liquid chromatography–tandem mass spectrometry (UPLC–MS, Thermo Fisher Ultimate–3000, Berlin, Germany) was used to qualitatively analyze the intermediate products of SMX. The separation was carried out with an MS detector and a Poroshell EC–C18 column (Agilent Technologies, Inc., Wilmington, DE, USA), (specification: 2.1 × 100 nm, particle size: 2.7 nm). The mobile phase was 0.1% formic acid: acetonitrile = 85:15 (volume ratio), the flow rate was 0.8 mL/min, and the injection volume was 50 μL. Electrospray ionization source (ESI) was used for mass spectrometry detection, the voltage was set to 30 V, and full scanning was carried out in positive ion mode, with the scanning range of mass-to-charge ratio (*m*/*z*) of 500–4500.

## 3. Structural Characterization Analysis of Molybdenite@BC

### 3.1. SEM–EDS Characterization Analysis

**Figure 1:**
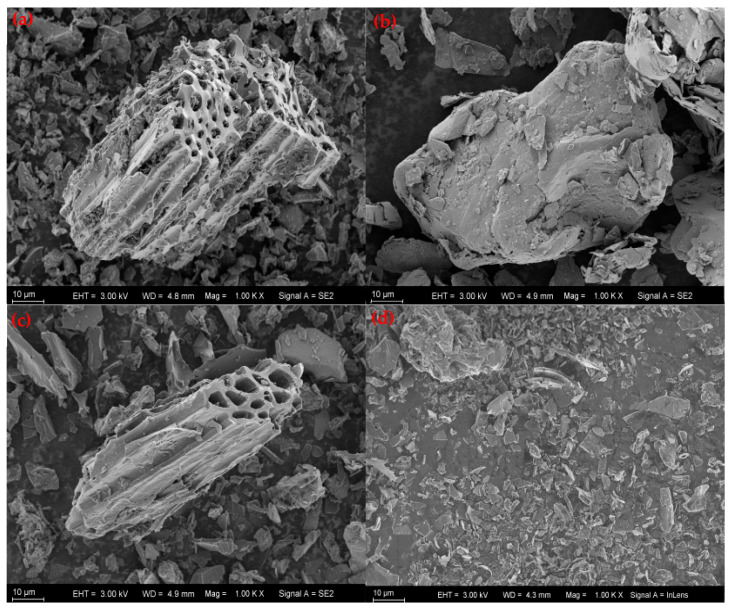
SEM images: BC samples (**a**); MoS_2_ samples (**b**); Molybdenite@BC (new) samples (**c**); Molybdenite@BC (used) samples (**d**).

The structure and morphology of BC, molybdenite, and finally selected Molybdenite@BC (new) and Molybdenite@BC (used) were analyzed by scanning electron microscope. As can be seen from Figure 1, BC had a porous structure, which was beneficial to the loading of Molybdenite. Molybdenite was mainly composed of Mo, S and other elements, which presented an irregular flaky structure. Molybdenite@BC (new) was a compact complex formed by molybdenite particles distributed on the surface or pores of BC. The results showed that there was local metal grain aggregation, and it could be observed that molybdenum-based oxide particles with different sizes were embedded in the pores of BC, which might be caused by high temperature pyrolysis. A large number of metal particles were uniformly dispersed on the surface and pores of BC, which was beneficial to electron transport in the catalytic process. In addition, the distribution of metal particles on the surface of Molybdenite@BC (used) was finer and more uniform, which showed that a small part of metal particles was dissolved in the reaction process and that MoS_2_ was stably loaded on the surface of BC. The results of energy dispersive spectrometer (EDS) showed that there were elements of C, S and Mo in the structure of Molybdenite@BC, and the composite was mainly composed of C (41.29 wt%, 75.40 at%), S (24.54 wt%, 16.79 at%) and Mo (34.17 wt%, 7.81 at%).

### 3.2. FT–IR Characterization Analysis

**Figure 2:**
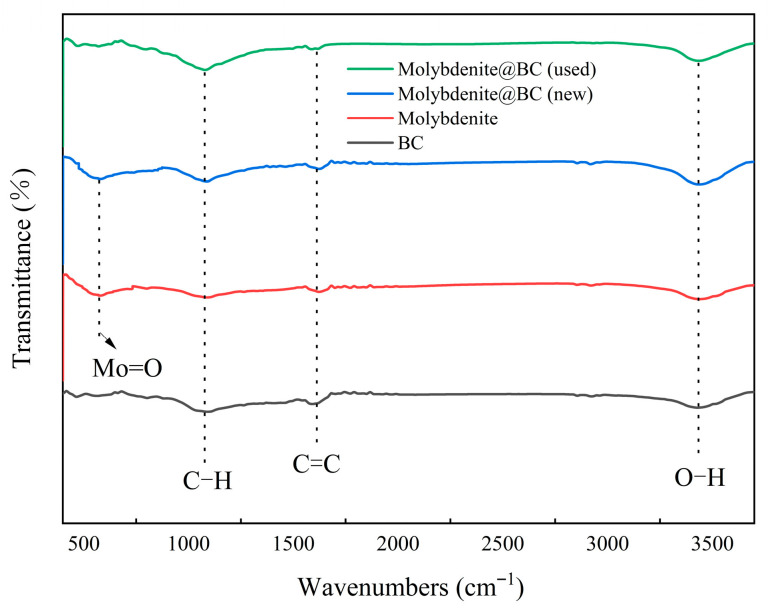
The FT–IR plots of the different materials.

Fourier infrared spectroscopy was applied in the characteristic region of 500–4500 cm^−1^, and the results are shown in Figure 2. Comparing four different materials, the characteristic absorption peaks at 1053.425 cm^−1^, 1609.306 cm^−1^ and 3434.118 cm^−1^ belonged to the tensile fluctuations of C–H, C=C and –OH respectively [31]. In addition, molybdenite and Molybdenite@BC (new) showed a new Mo=O stretching vibration peak at 580.476 cm^−1^ [32], and this new absorption peak indicated that Molybdenite@BC (new) contained molybdenum oxide. The appearance of Mo=O group proved that molybdenite was successfully introduced into the composites.

### 3.3. BET Characterization Analysis

In the experiment, four materials BC, molybdenite, Molybdenite@BC (new) and Molybdenite@BC (used) were tested by BET, and the parameters of BET specific surface area, average pore diameter and total pore volume of different materials obtained are shown in Table 1.

As can be seen from Table 1, the specific surface areas of BC, molybdenite and Molybdenite@BC (new) were 72.3758 m^2^/g, 2.9189 m^2^/g and 71.1043 m^2^/g respectively. The specific surface area of Molybdenite@BC (new) was lower than that of BC, which was due to the fact that part of molybdenite was filled into the pores of BC during the preparation of composite materials, resulting in the decrease in specific surface area. The specific surface area of Molybdenite@BC (used) was 195.8082 m^2^/g, which was much higher than that of the other three materials. This might be due to the chemical reaction of molybdenite during the reaction, which produced new oxides, thus increasing its specific surface area.

### 3.4. XRD Characterization Analysis

**Figure 3:**
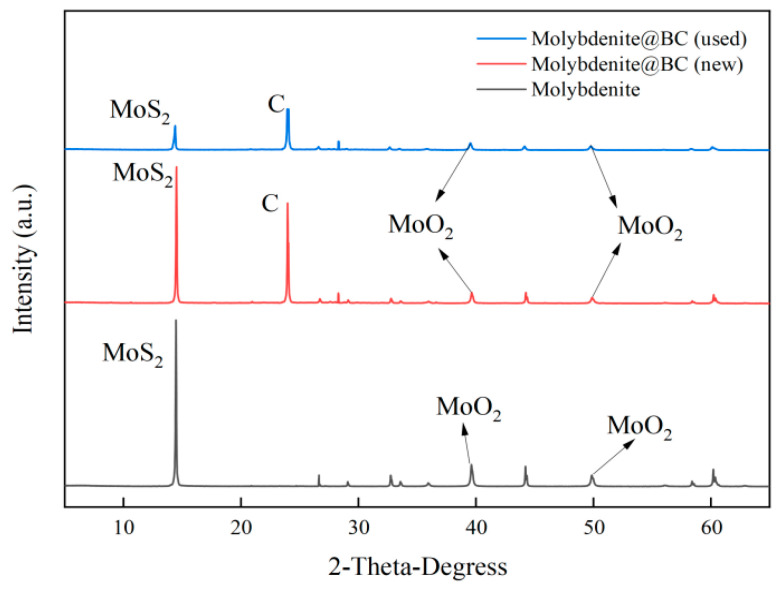
The XRD plots of the different materials.

The XRD characterization results of BC, molybdenite, Molybdenite@BC (new) and Molybdenite@BC (used) are shown in Figure 3. The results showed that after the introduction of molybdenite, the XRD patterns of Molybdenite@BC (new) and Molybdenite@BC (used) both showed characteristic peaks belonging to MoO_2_ at 2θ of about 39.65° and 51.87° [33]. The characteristic peak of MoS_2_, which was consistent with molybdenite, was observed at 2θ of about 14.29° [34]. The intensity of the characteristic peak corresponding to MoS_2_ in Molybdenite@BC (new) was weakened to some extent, which might be because that Molybdenite@BC (new) was prepared by compounding BC and molybdenite, then its MoS_2_ content was slightly lower than that of molybdenite. After the composite material was used, the peaks of MoO_2_ and MoS_2_ became weaker, which indicated that there was some loss of molybdenum from the material structure.

### 3.5. XPS Characterization Analysis

**Figure 4:**
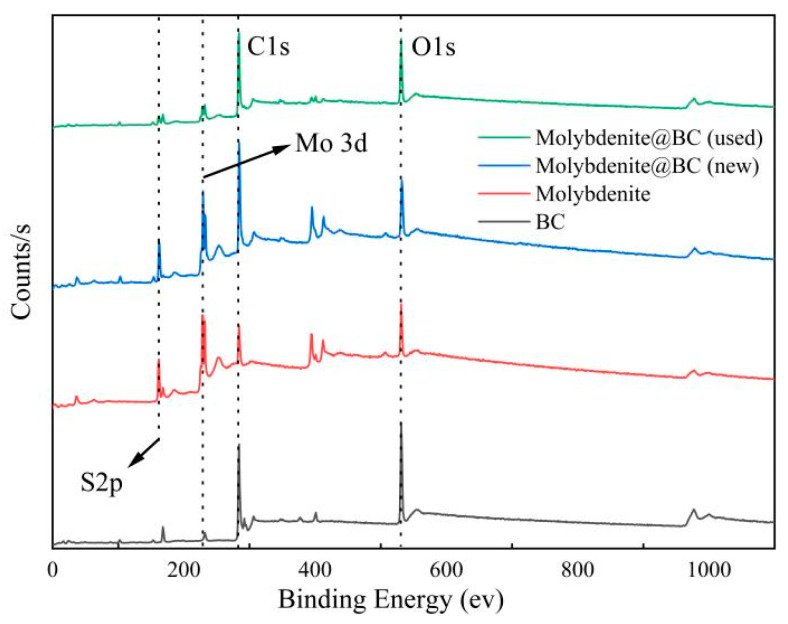
The XPS plots of the different materials.

In this experiment, four materials BC, molybdenite, Molybdenite@BC (new) and Molybdenite@BC (used) were analyzed by XPS, and the results are shown in Figure 4. The results showed that BC samples were mainly composed of C and O elements, and the binding energies of C1s and O1s were 284.08 eV and 516.63 eV, respectively, indicating that there were a large number of Oxygen-containing functional groups, such as carboxyl hydroxyl groups and ester groups in BC. Molybdenite samples contained Mo beside C and O elements, and the binding energy of Mo3d was 233.08 eV [35]. The elemental composition of Molybdenite@BC (new) sample was similar to that of molybdenite sample, but the binding energy of Mo3d was slightly reduced, indicating that there was some interaction between molybdenite and BC.

## 4. Single-Factor Experiment on SMX Degradation Efficiency by the Molybdenite@BC/PMS Process

**Figure 5:**
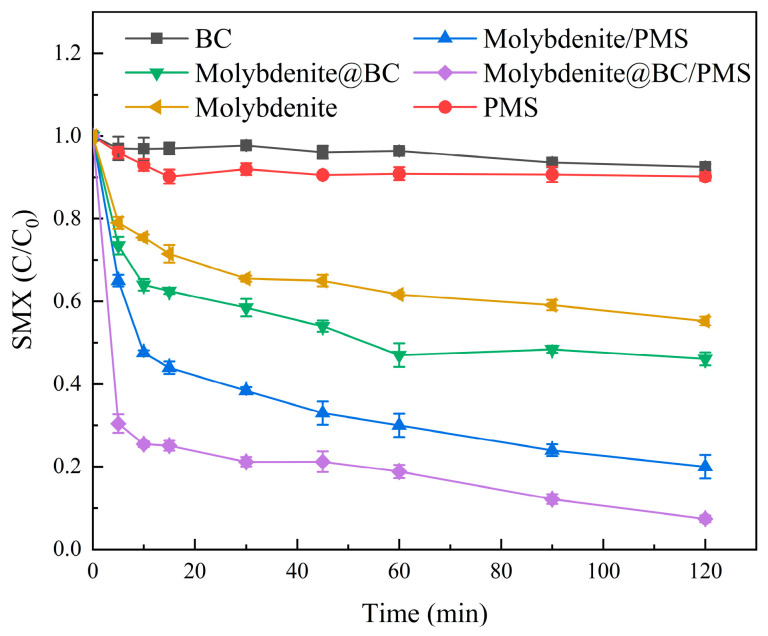
Degradation results of SMX in different systems.

In order to investigate the catalytic performance of Molybdenite@BC in the process of PMS activation, six oxidation systems were set up, namely, single PMS, single BC, molybdenite, Molybdenite/PMS, Molybdenite@BC, and Molybdenite@BC/PMS. The concentration of SMX in the above systems was set to 6 mg/L. The concentrations of PMS were 0.15, 0, 0, 0.15, 0, and 0.15 mM, respectively. The dosages of catalytic materials were 0, 0, 10, 10, 10, and 10 mg, respectively. The degradation results are shown in Figure 5.

The results in Figure 5 showed that there were differences in the degradation of SMX by the six different systems. In a single BC system, within 120 min, the porous structure of BC could remove SMX by physical adsorption, but the adsorption efficiency was limited, which proved that it was difficult to meet the requirements of pollutant removal by using BC alone. The Molybdenite system achieved 40% SMX removal rate. The reason might be that the layered structure of MoS_2_ contained unsaturated S sites, and SMX could be adsorbed by electrostatic action or π–π bond. However, the removal rate was much lower than that of the catalytic system with PMS, which showed that the core function of molybdenum was to activate PMS rather than degrade SMX alone. Compared with Molybdenite/PMS and Molybdenite@BC/PMS, the degradation rate of the latter in 120 min (>98%) was significantly higher than that of the former (≈75%). This was mainly due to the synergy of BC. On the one hand, BC provided a carrier for molybdenite to avoid its agglomeration and increased the active site of PMS. On the other hand, BC adsorbed SMX to the vicinity of the Molybdenite active site, enhancing its contact with SMX. Furthermore, the oxygen-containing functional groups on BC surface could also promote electron transfer and strengthen the generation of active oxygen free radicals. On the contrary, in the absence of BC, the catalytic efficiency of molybdenite was limited due to agglomeration.

### 4.1. Effect of Composite Materials with Different Mass Ratios on Degradation of SMX in Water

**Figure 6:**
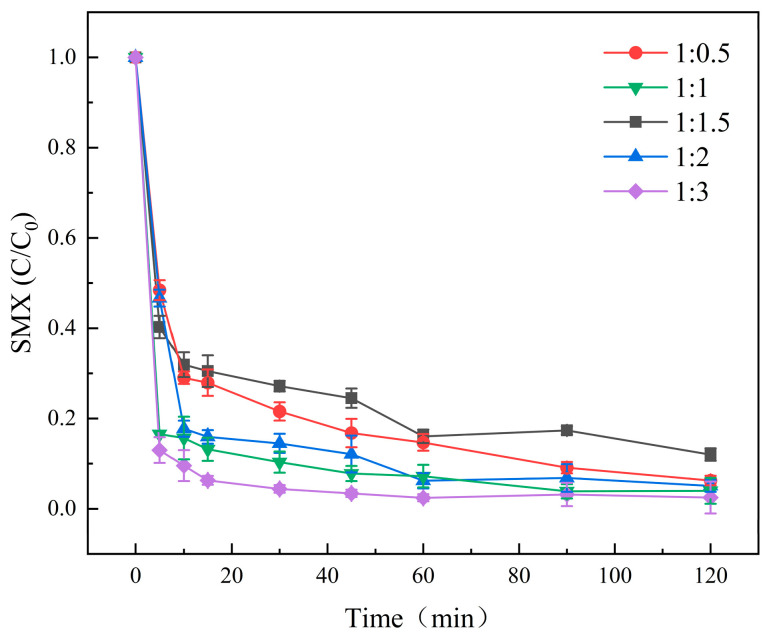
Degradation results of SMX under different composite ratio conditions. Aqueous phase temperature: 25 °C, Molybdenite@BC material dosage: 100 mg/L, [SMX]_0_ = 6 mg/L, [PMS]_0_ = 0.2 mM, pH = 6.9 ± 0.2, composite ratios of molybdenite and BC: 1:0.5, 1:1, 1:1.5, 1:2, and 1:3.

In this study, the effects of composite materials with different mass ratios on the degradation efficiency of SMX were investigated, and the results are shown in Figure 6. This figure shows that after 30 min of reaction, the degradation rates of SMX by Molybdenite@BC composites with composite ratios of 1:0.5, 1:1, 1:1.5, 1:2 and 1:3 were 68.2%, 85.7%, 62.1%, 79.4% and 93.1%, respectively. After 120 min of reaction, the degradation rates of SMX by Molybdenite@BC composites with composite ratios of 1:0.5, 1:1, 1:1.5, 1:2 and 1:3 were 94.49%, 97.24%, 88.96%, 95.85% and 98.69%, respectively. By comparison, it was found that the activated material with the mass ratio of 1:3 had the best degradation effect on SMX, which might be due to its high molybdenum content, thus providing more active molybdenum sites [36]. Therefore, the mass ratio of 1:3 was adopted in the subsequent experiments.

### 4.2. Effect of Composite Materials with Different Pyrolysis Temperatures on Degradation of SMX in Water

**Figure 7:**
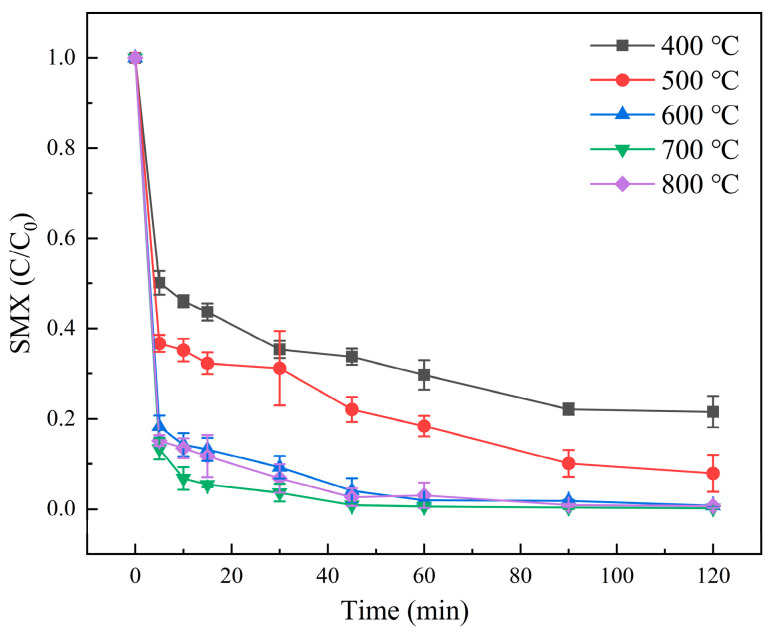
Results of SMX degradation at different pyrolysis temperatures. Aqueous phase temperature: 25 °C, Molybdenite@BC material dosage: 100 mg/L, [SMX]_0_ = 6 mg/L, [PMS]_0_ = 0.2 mM, pH = 6.9 ± 0.2, composite ratios of molybdenite and BC 1:3, pyrolysis temperatures: 400 °C, 500 °C, 600 °C, 700 °C, and 800 °C.

The existing research results show that the degradation effect of composite materials with different pyrolysis temperatures was different [37]. Therefore, the effects of Molybdenite@BC composites prepared at five different pyrolysis temperatures on the degradation efficiency of SMX were investigated, and the results are shown in Figure 7.

Figure 7 shows that after 30 min of reaction, the degradation rates of SMX in water by Molybdenite@BC with pyrolysis temperatures of 400 °C, 500 °C, 600 °C, 700 °C and 800 °C were 63.32%, 74.63%, 91.49%, 93.14% and 97.18%, respectively. After 120 min, the degradation rates of SMX were 78.89%, 89.23%, 98.45%, 98.64% and 98.08%, respectively. The experimental results showed that the total amounts of pollutants degraded by Molybdenite@BC materials prepared at 600 °C, 700 °C and 800 °C, respectively, were almost the same. However, Molybdenite@BC composites with a pyrolysis temperature of 700 °C within 30 min before the reaction have the highest degradation rate of SMX. Meanwhile, related research showed that excessive pyrolysis can lead to partial agglomeration [37], which will affect the activity of composites and reduce the degradation rate in the early stage of reaction. Therefore, the materials prepared at the temperature of 700 °C were used in the subsequent experiments.

### 4.3. Effect of Different Reaction Conditions on Degradation of SMX in Water

#### 4.3.1. Effect of Molybdenite@BC Dosage on SMX Degradation

**Figure 8:**
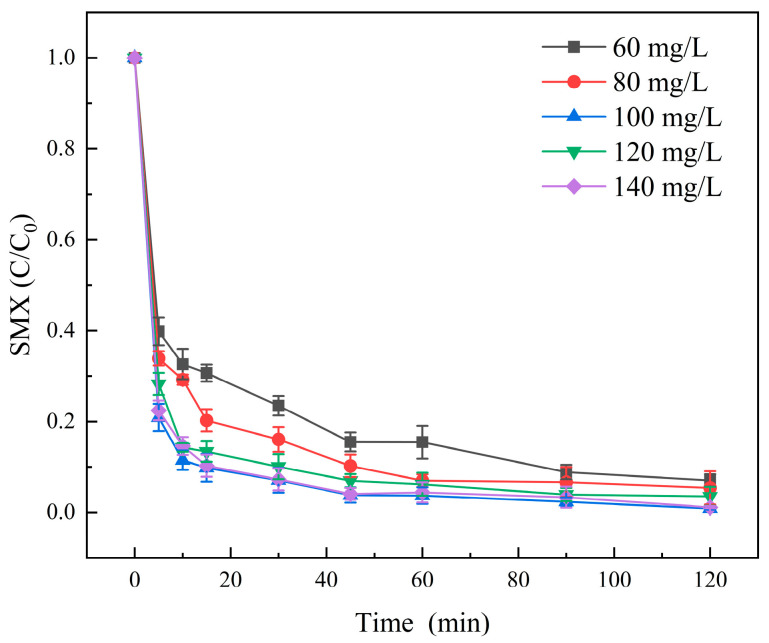
Degradation results of SMX under different material dosage conditions. Aqueous phase temperature: 25 °C, [SMX]_0_ = 6 mg/L, [PMS]_0_ = 0.2 mM, pH = 6.9 ± 0.2, Molybdenite@BC material dosage: 60, 80, 100, 120, and 140 mg/L.

In this study, the effect of the dosage of Molybdenite@BC on the degradation efficiency of SMX was investigated. The experimental results are shown in Figure 8. The figure shows after reaction for 120 min, when the dosage of Molybdenite@BC was 60, 80 and 100 mg/L, the degradation rates of SMX were 93.92%, 97.16% and 99.06%, respectively, and the overall trend was gradually increasing. However, with the gradual increase in the dosage of Molybdenite@BC, that was, when the dosage of the material was 120 and 140 mg/L, the degradation rate of SMX were 98.68% and 98.89%, respectively. The degradation rates showed a small downward trend. The above experimental phenomena showed that when the dosage of Molybdenite@BC was 100 mg/L, the best degradation effect was achieved. Therefore, it was meaningless to add Molybdenite@BC too much. The reason for this phenomenon might be that when the dosage of Molybdenite@BC material was too large, some materials would reunite in the solution and not completely diffuse into the reaction container, thus leading to a small decline in degradation efficiency. Therefore, the material dosage of 100 mg/L was used in the subsequent experiments.

#### 4.3.2. Effect of PMS Concentration on Degradation Efficiency of SMX

**Figure 9:**
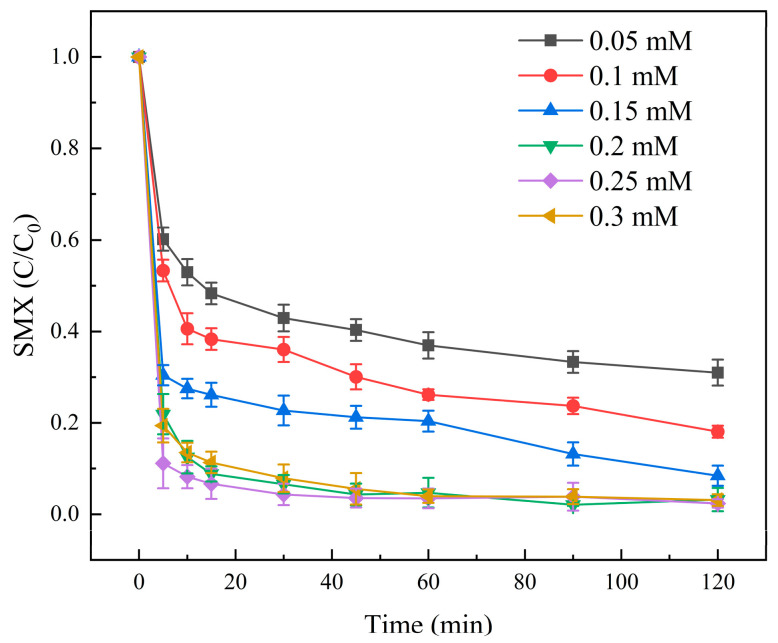
Degradation results of SMX at different PMS concentrations. Aqueous phase temperature: 25 °C, Molybdenite@BC material dosage: 100 mg/L, [SMX]_0_ = 6 mg/L, pH = 6.9 ± 0.2, PMS concentration: 0.05, 0.1, 0.15, 0.2, 0.25, and 0.3 mM.

In this study, the specific effect of PMS concentration on SMX degradation was investigated. The experimental results were shown in Figure 9. The results showed that the degradation rate of SMX increased continuously when the dosage of PMS was increased from 0.05 mM to 0.2 mM. However, when the concentration of PMS was continuously increased from 0.2 mM to 0.3 mM, the degradation rate of SMX remained almost unchanged after 60 min. It can be seen that the degradation effect of SMX was the best when the concentration of PMS was 0.2 mM, and the degradation rate of SMX after 60 min was 97.87%. Therefore, the degradation rate of SMX was not directly proportional to the dosage of PMS, so it was necessary to add an appropriate PMS concentration to make the degradation effect of the reaction system optimal. In the Molybdenite@BC/PMS reaction system, when the dosage of PMS was more than 0.2 mM, the amount of SMX degraded was almost the same as that of 0.2 mM. This was because when the concentration of PMS was too high, the oxidant itself would quench with free radicals. Therefore, with the decrease in free radicals, the degradation efficiency of pollutants would also decrease. At the same time, excessive PMS would be activated by activating materials to produce a large amount of SO_4_^•−^, and the SO_4_^•−^ would quench each other, which would make the free radicals fail to work, thus leading to the decline of the SMX degradation efficiency. Therefore, the reasonable dosage of PMS in this experiment was 0.2 mM.

#### 4.3.3. Effects of Different pH Values on the Degradation of SMX

**Figure 10:**
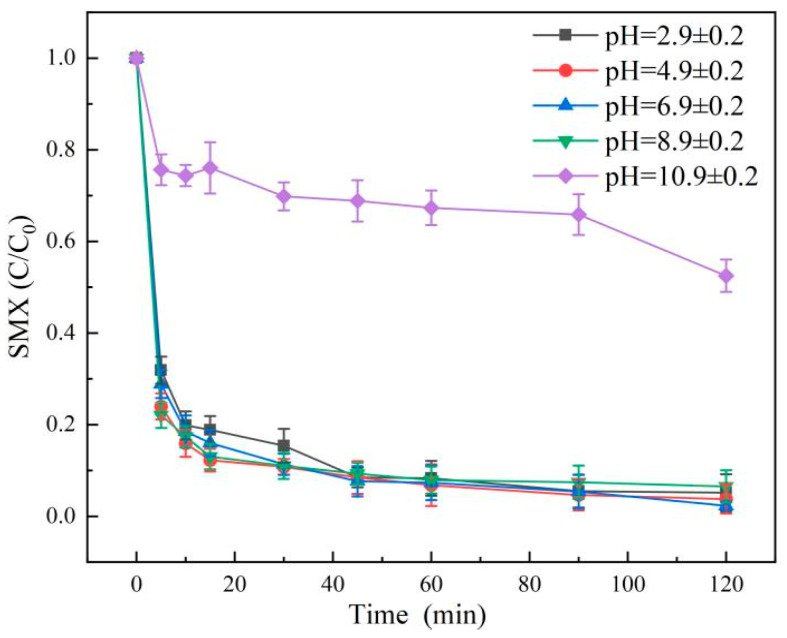
Results of SMX degradation at different pH. Aqueous phase temperature: 25 °C, Molybdenite@BC material dosage: 100 mg/L, [SMX]_0_ = 6 mg/L, [PMS]_0_ = 0.2 mM, pH value: 2.9 ± 0.2, 4.9 ± 0.2, 6.9 ± 0.2, 8.9 ± 0.2, and 10.9 ± 0.2.

The degradation results of SMX under different pH conditions are shown in Figure 10. The results showed that after 120 min of reaction, the degradation rates of SMX were 97.73%, 98.48%, 98.41% and 96.07% when the pH values were 2.9 ± 0.2, 4.9 ± 0.2, 6.9 ± 0.2 and 8.9 ± 0.2, respectively. It can be seen that the degradation rates of SMX were similar under the above reaction conditions. However, the experimental results showed that the degradation efficiency of SMX began to decrease significantly when pH continued to increase to strong alkalinity. Therefore, when the reaction system was acidic, neutral and weakly alkaline, PMS could be effectively catalyzed to produce SO_4_^•−^, and at the same time, part of S_2_O_8_^2−^ reacted with H^+^ to produce SO_4_^•−^, so the degradation rate of SMX was accelerated, and the system had a wider pH range. In addition, the inhibition of the reaction was intensified under strong alkaline conditions. When the pH was greater than 8.9 ± 0.2, ^•^OH would react with OH^−^ in the system to generate ^•^O_2_^−^, and the oxidation activity of ^•^O_2_^−^ on SMX was much lower than that of ^1^O_2_. Meanwhile, high concentration of OH^−^ would also promote the transformation of SO_4_^•−^ to ^•^OH, and excessive ^•^O_2_^−^ would quench with ^1^O_2_, resulting in a significant decrease in the concentration of active species (^1^O_2_) leading to degradation. In summary, the degradation rate dropped sharply at pH = 10.9, which was essentially the synergistic effect of electrostatic repulsion, SMX adsorption inhibition and reactive oxygen species (ROS) transformation path change caused by surface charge inversion between PMS and molybdenite. This provided clear mechanism support for the pH application range (acidic to weakly alkaline) of the system [22].

### 4.4. Effect of Inorganic Anions on Degradation of SMX in Water

**Figure 11:**
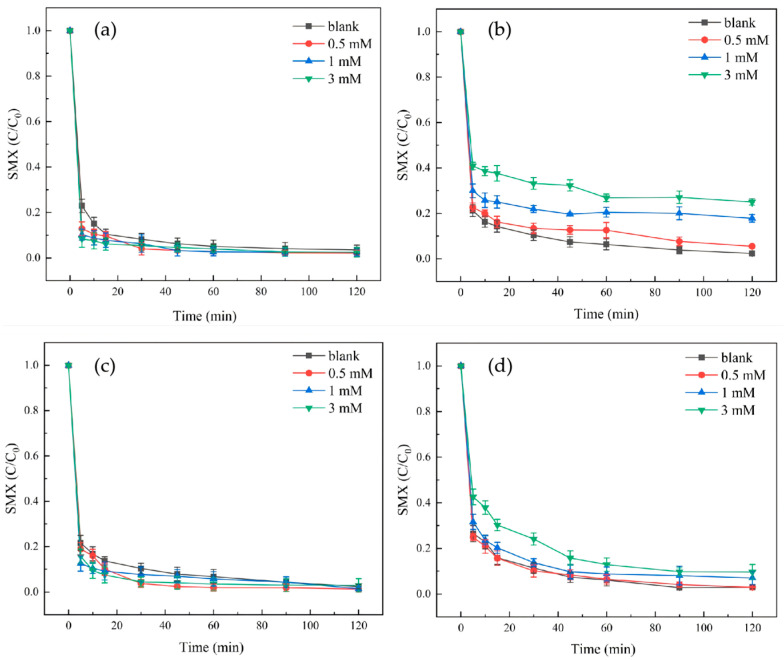
Effect of different concentrations of inorganic anions on SMX degradation. (**a**) Cl^−^; (**b**) HCO_3_^−^; (**c**) SO_4_^2−^; (**d**) HA. Reaction condition: aqueous phase temperature 25 °C, Molybdenite@BC material dosage 100 mg/L, [SMX]_0_ = 6 mg/L, [PMS]_0_ = 0.2 mM, pH = 6.9 ± 0.2.

Different kinds of anions in the water environment may have different degrees of inhibition on the reaction system. Therefore, in this experiment, Cl^−^, HCO_3_^−^, SO_4_^2−^ and humic acid (HA), which are common in groundwater, were selected as representatives to investigate the influence of inorganic anions in groundwater on the degradation of SMX by the Molybdenite@BC/PMS system. The experiment was carried out at 25 °C, the initial concentration of SMX was 6 mg/L, the initial concentration of PMS was 0.2 mM, the dosage of Molybdenite@BC was 100 mg/L, and the initial pH was 6.9 ± 0.2. The concentrations of anions were controlled at 0.5, 1 and 3 mM, respectively. The blank group had the same conditions as the experimental group except that no anions were added. The effects of different concentrations of inorganic anions (0.5, 1, and 3 mM) on the degradation of SMX in the Molybdenite@BC/PMS system are shown in Figure 11.

As shown in Figure 11a, the results showed that with the increase in concentration, Cl^−^ promoted the degradation of SMX in the Molybdenite@BC/PMS system in a certain concentration range. Cl^−^ could react with the original free radicals in the system to generate new oxidizing species. As an electron donor, Cl^−^ could react with SO_4_^•−^ (redox potential 2.5–3.1 V) and ^•^OH (redox potential 1.8–2.7 V) to generate Cl^•^ (redox potential 2.4 V) and Cl_2_^•−^ (redox potential 2.0 V) and other chlorine-based free radicals. Although the oxidation activity of these chlorine-based radicals was slightly lower than that of SO_4_^•−^, they had a longer half-life (Cl^•^ ≈ 10^−9^ s, SO_4_^•−^ ≈ 10^−10^ s), and their diffusion range in aqueous solution was wider, so they could contact with SMX molecules more efficiently. In addition, Cl_2_^•−^ could further generate ClO^•^ and other species through chain reaction, which would continue to participate in the oxidative degradation of SMX and improve the oxidation ability of ROS in the system. Therefore, when the Molybdenite@BC/PM system was used to degrade SMX in water, the influence of Cl^−^ concentration should be considered to achieve the best degradation effect.

The effect of HCO_3_^−^ on the degradation of SMX in the Molybdenite@BC/PMS system at different concentrations is shown in Figure 11b. The experimental results showed that the existence of HCO_3_^−^ had a significant effect on the degradation efficiency of SMX in the Molybdenite@BC/PMS system. Meanwhile, the increase in HCO_3_^−^ concentration could inhibit the degradation efficiency of SMX. The reason might be that HCO_3_^−^ reacted with the free radicals generated by the Molybdenite@BC/PMS system to form stable carbonate free radicals, thus reducing the oxidation of SMX. In addition, HCO_3_^−^ might form a stable complex with Mo^2+^, thus reducing the effective concentration of Mo^2+^, and further affecting the activation of PMS.

The effects of SO_4_^2−^ at different concentrations on the degradation of SMX by Molybdenite@BC/PMS are shown in Figure 11c. The figure shows that in the Molybdenite@BC/PMS system, SO_4_^2−^ might have an enhancing effect on the degradation of SMX, but that the results were inconclusive.

The effects of HA at different concentrations on the degradation of SMX by Molybdenite@BC/PMS are shown in Figure 11d. The experimental results showed that with the increase in HA concentration, the degradation efficiency of SMX decreased more and more obviously. The inhibition of HA on degradation mainly came from two aspects. The first was the competitive consumption of active species. As a macromolecular organic substance rich in phenolic hydroxyl and carboxyl, HA could compete with SMX for active species in the system. The reaction rate constant of HA and ^1^O_2_ (k ≈ 1.0 × 10^8^~5.0 × 10^8^ M^−1^s^−1^) was close to that of SMX (k = 1.2 × 10^8^ M^−1^s^−1^), which would consume part of ^1^O_2_ through oxidative degradation (such as benzene ring opening and functional group transformation), resulting in a decrease in the concentration of active species involved in SMX degradation. At the same time, HA would also react with ^•^OH and SO_4_^•−^ (k was 1.0 × 10^9^~1.0 × 10^10^ and 1.0 × 10^8^~1.0 × 10^9^ M^−1^s^−1^ respectively), further weakening the oxidation capacity. The second was the occupation of the active site of the catalyst. Polar functional groups (–COOH, –OH) of HA could be adsorbed on the surface of Molybdenite@BC through hydrogen bonding and electrostatic interaction, covering the Molybdenite active site (Mo^4+^/Mo^6+^) and porous structure of BC. This result not only hindered the activation of PMS at the active site, but also reduced the adsorption of pollutants on the surface of the material [38].

## 5. Mechanism and Degradation Products Analysis of SMX in Water by Molybdenite@BC/PMS

### 5.1. Study on the Mechanism of Degradation of SMX by the Molybdenite@BC/PMS System

#### 5.1.1. Removal Rate of Total Organic Carbon (TOC) in the Degradation of SMX by the Molybdenite@BC/PMS System

In order to further analyze the reaction mechanism of the Molybdenite@BC/PMS system, electron paramagnetic resonance (EPR), free radical quenching experiment and TOC removal rate were used to identify the active species of the system. UPLC–MS/MS was used to infer the intermediate products and degradation paths of SMX.

**Figure 12:**
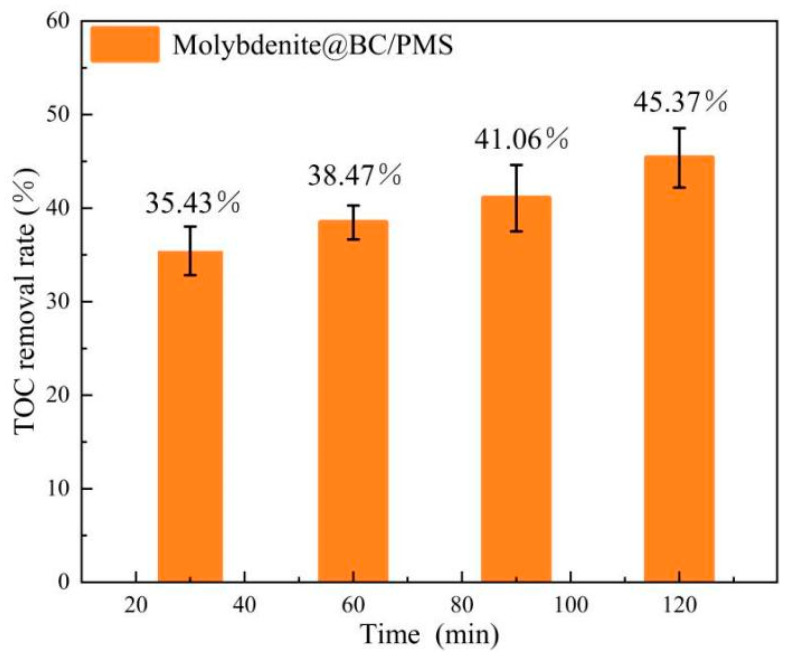
TOC removal rate of Molybdenite@BC/PMS system at different degradation times.

Under the conditions of temperature 25 °C, dosage of Molybdenite@BC 100 mg/L, initial concentration of SMX 6 mg/L, dosage of PMS 0.2 mM and pH = 6.9 ± 0.2, the mineralization rate of the Molybdenite@BC/PMS system for SMX in water was investigated. The experimental results are shown in Figure 12. The results showed that the mineralization rates of the Molybdenite@BC/PMS system for SMX in water were 35.43%, 38.47%, 41.06% and 45.37%, respectively, at different reaction times.

The experimental results showed that the Molybdenite@BC/PMS system could achieve about 95% SMX degradation within 30 min, but the TOC removal rate was only 35.43% in this reaction time. When the reaction time was 60 min, the degradation rate of SMX increased to 97.8%, and the mineralization degree was higher than that at 30 min. The remarkable degradation efficiency of the system at 30 min might be attributed to two mechanisms: one was the direct adsorption of SMX by Molybdenite@BC; secondly, the catalyst promoted the generation of free radicals and improved their utilization efficiency, thus accelerating the mineralization of some SMX.

#### 5.1.2. Free Radical Quenching Experiment and Analysis of the Molybdenite@BC/PMS System

**Figure 13:**
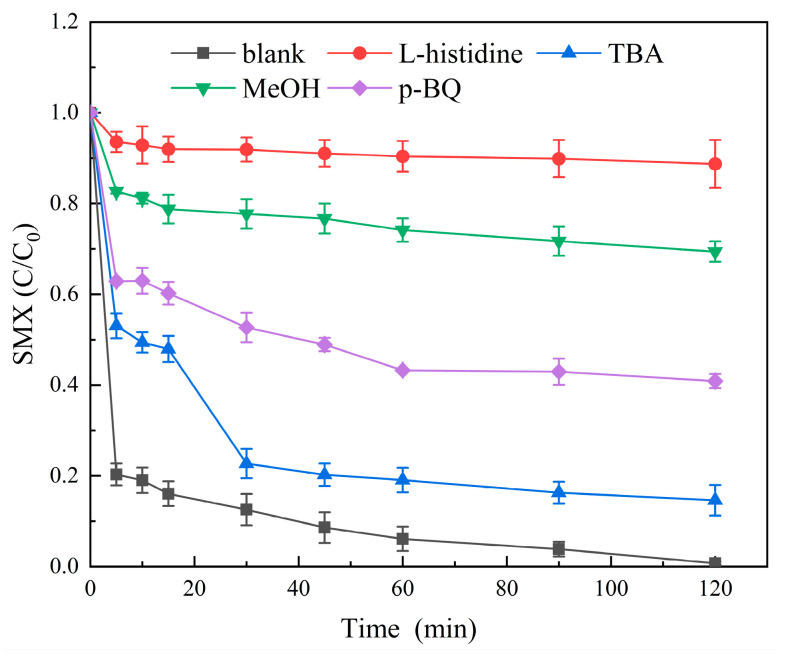
Effect of different quenchers on the Molybdenite@BC/PMS system.

In order to clarify the composition and contribution rate of active species in the Molybdenite@BC/PMS system, a free radical quenching experiment was used in this study. Tert–butanol (TBA) and methanol (MeOH) were selected as quenching agents for ^•^OH and SO_4_^•−^, while L–histidine and p–benzoquinone (p–BQ) were used to quench singlet oxygen (^1^O_2_) and superoxide radical (^•^O_2_^−^). The experiment was carried out at the reaction temperature of 25 °C, the dosage of Molybdenite@BC was 100 mg/L, the initial concentration of SMX was 6 mg/L, the dosage of PMS was 0.2 mM, the pH was 6.9 ± 0.2, and the dosage of all quenchers was 100 mM. The quenching of various free radicals by the Molybdenite@BC/PMS system was investigated. The effects of different quenchers on the Molybdenite@BC/PMS system were shown in Figure 13.

Figure 13 shows that compared with the blank control group, after adding 100 mM L–histidine, the degradation effect of the Molybdenite@BC/PMS system on SMX in water was obviously weakened, and the degradation rate was only 18.9%, which indicated that ^1^O_2_ played an important role in the degradation process of SMX. Meanwhile, adding MeOH to the Molybdenite@BC/PMS system could further reduce the degradation rate of SMX compared with adding TBA, which indicated that SO_4_^•−^ could effectively remove SMX from water. In addition, the data showed that TBA had the least influence on the SMX degradation rate, which might be because ^•^OH was not the dominant factor in the degradation process of SMX. After adding MeOH and TBA, the SMX degradation rate by Molybdenite@BC/PMS decreased from 98.64% to 32.16% and 87.74%, respectively. It could be inferred that the contribution rate of SO_4_^•−^ in the Molybdenite@BC/PMS system was greater than that of ^•^OH. After adding p–BQ, the SMX degradation rate by Molybdenite@BC/PMS decreased from 98.64% to 60.21%, indicating that the contribution rate of ^•^O_2_^−^ in this system was greater than that of ^•^OH. Therefore, it could be seen that the dominant free radicals in the Molybdenite@BC/PMS system could be inferred by comparing the effects of different quenchers on the degradation rate of SMX.

#### 5.1.3. Analysis of Free Radicals in the Molybdenite@BC/PMS System for Degradation of SMX

**Figure 14:**
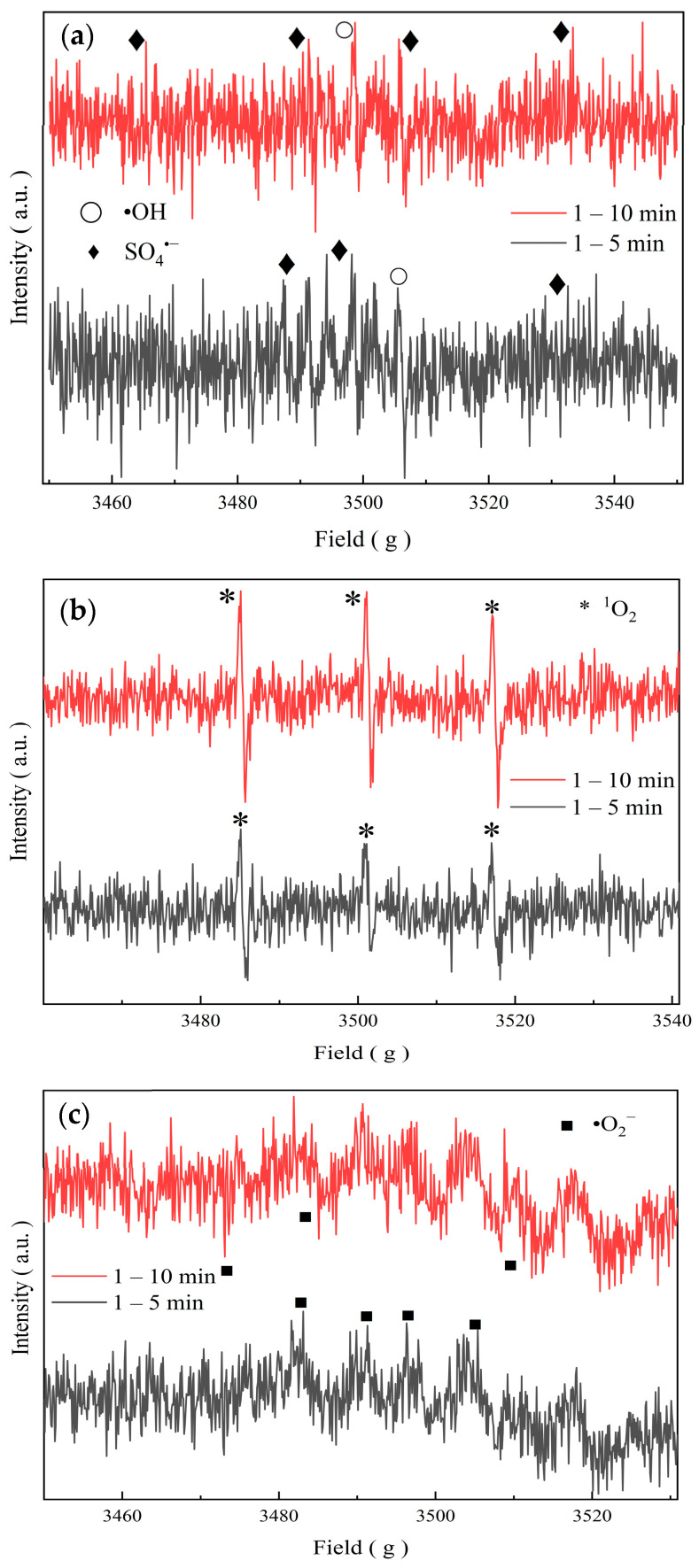
Plot for EPR detection profiles. (**a**) DMPO–^•^OH, DMPO–SO_4_^•−^; (**b**) DMPO–^•^O_2_^−^; (**c**) TEMP–^1^O_2_.

In this study, EPR was used to identify the active species in the Molybdenite@BC/PMS system, and DMPO and TEMP were selected as spin trapping agents. Spin trapping agent can generate stable spin adducts with active species, and its EPR spectrum has characteristic signals. DMPO can effectively capture ^•^OH, SO_4_^•−^, and ^•^O_2_^−^, and then generate three characteristic adducts, namely, DMPO–^•^OH, DMPO–SO_4_^•−^, and DMPO–^•^O_2_^−^. TEMP, as a capture agent for detecting ^1^O_2_, can generate stable TEMP–^1^O_2_ adduct [30]. The EPR detection spectrum is shown in Figure 14.

It can be seen in Figure 14 that the adduct of TEMP–^1^O_2_ was observed in the Molybdenite@BC/PMS system by EPR analysis, indicating the existence of ^1^O_2_ as a non-free radical in this system (g Field: 3486, 3502 and 3518). In addition, the signal intensities of the corresponding adduct of ^•^OH, SO_4_^•−^, and ^•^O_2_^−^ were relatively weak, which indicated that the amount of these free radicals were small during the reaction. Figure 14c shows that the peak area of TEMP–^1^O_2_ also increased with the passage of time, which was consistent with the results of the previous free radical quenching experiment, indicating that ^1^O_2_ was dominant in the Molybdenite@BC/PMS system [36]. The above results showed that not only ^•^OH, SO_4_^•−^, and ^•^O_2_^−^ existed in the Molybdenite@BC/PMS system, but also ^1^O_2_ in the form of non-radicals, furthermore, ^1^O_2_ was dominant in this system.

### 5.2. Analysis of SMX and Intermediate Products

#### 5.2.1. SMX Intermediate Products

According to UPLC–MS/MS, the intermediate products produced in the degradation process of SMX were inferred, and the information is shown in Table 2. In this process, nine kinds of intermediate products with mass-to-charge ratios of 270.0543, 266.0241, 282.0190, 298.0139, 176.9976, 201.9816, 225.9928, 239.0485 and 138.0197 were determined.

#### 5.2.2. Analysis of SMX Degradation Path

**Figure 15:**
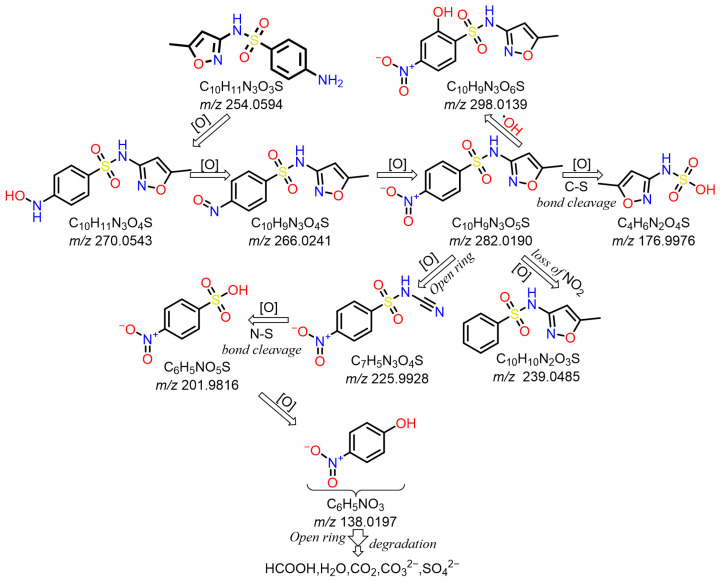
Possible degradation pathways of sulfamethoxazole.

The possible degradation pathway of SMX is shown in Figure 15. Firstly, protonated excimer ions will be generated from the initial substrate during mass spectrometry ionization, and its mass-to-charge ratio (*m*/*z*) was 254.0594. Based on these molecular structure analyses, three possible degradation pathways of SMX were proposed. In the first degradation path, the amino group of the initial substrate underwent oxidative hydroxylation reaction to produce hydroxylamine product **P1** (*m*/*z* = 270.0543) [39]. The hydroxylamine product **P1** was further oxidized and transformed into nitroso product **P2** (*m*/*z* = 266.0241). The nitroso product **P2** continued to be oxidized to form nitro product **P3** (*m*/*z* = 282.0190).

Nitro product **P3** had many further degradation pathways as follows: (1) hydroxyl radical attacked benzene ring, and product **P4** (*m*/*z* = 298.0139) was generated; (2) the C–S bond was oxidized and broken, resulting in **P5** (*m*/*z* = 176.9976) product N–methoxazole sulfonic acid [40,41]; (3) the oxazole ring was opened, and the product **P7** (*m*/*z* = 225.9928) was formed; (4) In addition, the nitro product **P3** could be denitrified to obtain the product **P8** (*m*/*z* = 239.0485) [42].

Further degradation of the product **P7**: the product **P7** would undergo oxidative cleavage of the N–S bond to obtain the product **P6** (*m*/*z* = 201.9816) p–nitrobenzenesulfonic acid [43]. **P6** was further stripped of SO_2_ to produce **P9** (*m*/*z* = 138.0197) p–nitrophenol [44].

The analysis results showed that the product underwent multi-step transformation in the process of oxidative degradation: firstly, the oxidative cracking of the ring occurred, resulting in small molecular organic acids and other substances; then, after deep mineralization, it was finally transformed into H_2_O, CO_2_, CO_3_^2−^, H_2_SO_4_ and other substances, realizing the complete degradation of the compound. In this process, ^1^O_2_ was the main reason for these conversion pathways.

By analyzing the structure and types of the above products, it can be inferred that the degradation of SMX mainly came from these different reaction channels. These reaction steps were interrelated and together constituted the degradation process of the compound. The accurate determination and structural identification of products in each stage will help to deeply understand the degradation mechanism of compounds under environmental or other conditions, and provide theoretical basis for further research and application.

#### 5.2.3. Toxicity ECOSAR Analysis of SMX and Intermediate Products

In this study, the toxicity of SMX and its nine intermediates was predicted by using ECOSAR v 2.2 (U.S. Environmental Protection Agency, Washington, DC, USA) software, and the results are shown in Table 3. Table 3 shows that the oxidation products **P1** (*m*/*z* = 270.0543), **P3** (*m*/*z* = 282.0190), **P4** (*m*/*z* = 298.0139) and **P5** (*m*/*z* = 176.9976) of SMX had high toxicity. Among them, the oxidation product **P1** of SMX continued to be oxidized to form **P2** (*m*/*z* = 266.0241), and the nitroso product **P2** continued to be oxidized to form nitro product **P3**, and the toxicity decreased greatly. The ^•^OH attacked the benzene ring of **P3**, forming a very weak toxic product **P4**; On the other side, the C–S bond breaks, resulting in the weakest toxic product **P5**. In addition, in the third pathway of **P3**, the oxazole ring of **P3** opened to form **P7** (*m*/*z* = 225.9928), and the toxicity of **P7** increased greatly. On the other hand, **P3** formed **P8** (*m*/*z* = 239.0485) through denitrification, and the toxicity of **P8** was greatly increased, probably because the structure of **P8** was more complicated than that of **P7**, and its bioavailability and bioaccumulation potential were higher, thus increasing the potential harm to organisms. **P6** (*m*/*z* = 201.9816) was obtained by oxidative cleavage of the N–S bond of **P7**, and the toxicity was reduced again. **P6** further removed SO_2_ to generate **P9** (*m*/*z* = 138.0197). The results showed that the overall toxicity of SMX decreased during the degradation process.

## 6. Conclusions and Future Research Direction

In this study, the efficiency, mechanism and intermediate products of Molybdenite@BC composite activating the PMS process for removing SMX from water were analyzed, and the following conclusions were obtained:

(1) Molybdenite@BC-activated material was prepared, characterized and analyzed. SEM–EDS results showed that Molybdenite@BC-activated materials displayed good reactivity, although there was some loss of molybdenum from the material structure. XRD analysis showed that molybdenite and BC mainly existed in the form of physical combination. The BET results indicated that Molybdenite@BC-activated material had a high specific surface area, which was beneficial to improve the adsorption efficiency. XPS and FT–IR analysis revealed the changes in chemical composition and functional groups of Molybdenite@BC-activated materials before and after use, and the formation of Mo=O bond in Molybdenite@BC (new) indicated the loading effect of molybdenite on BC.

(2) Compared with single PMS, single BC, molybdenite, Molybdenite/PMS, and Molybdenite@BC, Molybdenite@BC/PMS had the best degradation effect on SMX. When the pyrolysis temperature was 700 °C and the mass ratio of molybdenite to biochar was 1:3, the degradation rate reached 98.64%. In addition, the degradation rate of SMX could be improved by adding appropriate amounts of activated materials, and the optimal dosage was 100 mg/L. The experimental data showed that the degradation rate of SMX was the highest when the initial PMS concentration is 0.2 mM. Meanwhile, the degradation effect of SMX under neutral and acidic conditions was better than that under alkaline conditions. Common inorganic anions, such as HCO_3_^−^, could inhibit the SMX degradation, while appropriate amounts of Cl^−^ could promote the degradation. SO_4_^2−^ might have an enhancing effect on the SMX degradation, but the results were inconclusive. HA could inhibit the degradation of SMX in the Molybdenite@BC/PMS system, and the inhibition effect was gradually enhanced with the increase in HA concentration (0.5–3.0 mM).

(3) Through the free radical quenching and EPR experiment, the species and activities of reactive oxygen radicals that might exist in the reaction system were identified, including ^•^OH, ^1^O_2_, SO_4_^•−^, and ^•^O_2_^−^. In this process, ^1^O_2_ was the main reason for these conversion pathways. XPS results showed that molybdenum participated in the reaction, and promoted the degradation of SMX in the Molybdenite@BC/PMS system. The degradation products after the reaction were analyzed by LC–MS, and three possible degradation paths were put forward. The results of ECOSAR analysis showed that the overall toxicity tended to decrease during the degradation of SMX. Finally, SMX was mineralized into small molecular compounds, such as CO_2_ and H_2_O.

(4) On the basis of this study, there are still some aspects worthy of further study in the future, as follows: firstly, this experiment was only static, and the availability of this process to degrade SMX in the flow-through reactor has not been explored; secondly, the repeated batches that this process can handle have not been investigated. Furthermore, the leaching of molybdenum and the long-term stability of the composite need further investigation.

## Figures and Tables

**Table 1:** The BET specific surface area of the different materials.

Material Type	Specific Surface Area (m^2^/g)	Mean Pore Size (nm)	Total Pore Volume (cm^2^/g)
BC	72.38	2.91	0.05
Molybdenite	2.92	20.78	0.02
Molybdenite@BC (new)	71.11	16.57	0.06
Molybdenite@BC (used)	195.81	2.91	0.14

**Table 2:** SMX intermediate information.

Number	Mass-to-Charge Ratio	Structural Formula	Constitutional Formula
SMX	254.0594	C_10_H_11_N_3_O_3_S	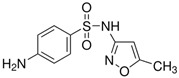
**P1**	270.0543	C_10_H_11_N_3_O_4_S	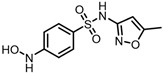
**P2**	266.0241	C_10_H_9_N_3_O_4_S	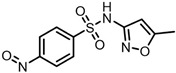
**P3**	282.0190	C_10_H_9_N_3_O_5_S	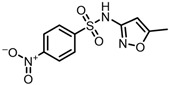
**P4**	298.0139	C_10_H_9_N_3_O_6_S	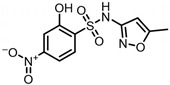
**P5**	176.9976	C_4_H_6_N_2_O_4_S	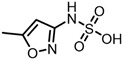
**P6**	201.9816	C_6_H_5_NO_5_S	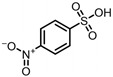
**P7**	225.9928	C_7_H_5_N_3_O_4_S	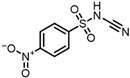
**P8**	239.0485	C_10_H_10_N_2_O_3_S	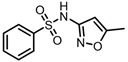
**P9**	138.0197	C_6_H_5_NO_3_	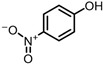

**Table 3:** Toxicity of SMX and its intermediates (predicted by ECOSAR).

Compound	Acute Toxicity (mg/L)			Chronic Toxicity (ChV) (mg/L)		
	Fish LC_50_ (96 h)	Daphnid LC_50_ (48 h)	Green Algae EC_50_ (96 h)	Fish LC_50_	Daphnid LC_50_	Green Algae EC_50_
SMX	329.561	34.163	56.214	42.07	38.679	169.201
**P1**	5554.786	2731.262	1122.098	1047.43	2364.945	23.533
**P2**	991.198	526.176	296.742	265.138	408.436	7.125
**P3**	11,987.005	47,954.203	11,443.553	11,514.4	50,081.801	190.308
**P4**	31,782.005	13,187.005	25,985.607	22,320.2	1438.358	9261.028
**P5**	47,487.128	74,888.719	25,887.293	86,578.6	30,145.912	29,511.659
**P6**	4886.424	1987.568	1478.153	2357.58	1238.612	975.651
**P7**	3841.489	1478.65	627.239	1287.5	324.598	784.127
**P8**	6675.488	362.909	215.072	190.854	276.924	156.274
**P9**	7369.818	3210.006	1088.433	2415.66	509.249	1108.048

## Data Availability

Data are contained within the article.

## References

[1] Zhang Y., Qin Y., Yang C., Gai T., Zhao Q., Xie J. (2025). Antibiotic distribution and ecological risk in the tropical waters of western Hainan: A comparative analysis of the dry and rainy seasons. Environ. Res..

[2] Jiang L., Zhai W., Wang J., Li G., Zhou Z., Li B., Zhuo H. (2023). Antibiotics and antibiotic resistance genes in the water sources of the Wuhan stretch of the Yangtze River: Occurrence, distribution, and ecological risks. Environ. Res..

[3] Qin L., Pang X., Zeng H., Liang Y., Mo L., Wang D., Dai J. (2020). Ecological and human health risk of sulfonamides in surface water and groundwater of Huixian karst wetland in Guilin, China. Sci. Total Environ..

[4] Archundia D., Duwig C., Spadini L., Morel M.C., Prado B., Perez M.P., Orsag V., Martins J.M.F. (2019). Assessment of the Sulfamethoxazole mobility in natural soils and of the risk of contamination of water resources at the catchment scale. Environ. Int..

[5] Prasannamedha G., Kumar P.S. (2020). A review on contamination and removal of sulfamethoxazole from aqueous solution using cleaner techniques: Present and future perspective. J. Clean. Prod..

[6] Miyake K., Kawamura T., Nakahara Y., Sasaki S. (2023). A single–center, person–month–based analysis of the risk of developing Pneumocystis pneumonia (PCP) in immunosuppressed non–HIV patients: Preventive effects of trimethoprim–sulfamethoxazole. J. Infect. Chemother..

[7] Passerini M., Nayfeh T., Yetmar Z.A., Coussement J., Goodlet K.J., Lebeaux D., Gori A., Mahmood M., Temesgen Z., Murad M.H. (2024). Trimethoprim–sulfamethoxazole significantly reduces the risk of nocardiosis in solid organ transplant recipients: Systematic review and individual patient data meta–analysis. Clin. Microbiol. Infect..

[8] Yang Y., Ok Y.S., Kim K., Kwon E., Tsang Y. (2017). Occurrences and removal of pharmaceuticals and personal care products (PPCPs) in drinking water and water/sewage treatment plants: A review. Sci. Total Environ..

[9] Carballa M., Omil F., Lema J.M., Llompart M., García–Jares C., Rodríguez I., Gómez M., Ternes T. (2004). Behavior of pharmaceuticals, cosmetics and hormones in a sewage treatment plant. Water Res..

[10] Carballa M., Omil F., Ternes T., Lema J.M. (2007). Fate of pharmaceutical and personal care products (PPCPs) during anaerobic digestion of sewage sludge. Water Res..

[11] Batt A.L., Kim S., Aga D.S. (2006). Enhanced biodegradation of iopromide and trime thoprim in nitrifying activated sludge. Environ. Sci. Technol..

[12] Huang S., Bao J., Shan M., Qin H., Wang H., Yu X., Chen J., Xu Q. (2018). Dynamic changes of polychlorinated biphenyls (PCBs) degradation and adsorption to biochar as affected by soil organic carbon content. Chemosphere.

[13] Tonucci M.C., Gurgel L.V.A., Aquino S.F.D. (2015). Activated carbons from agricultural byproducts (pine tree and coconut shell), coal, and carbon nanotubes as adsorbents for removal of sulfamethoxazole from spiked aqueous solutions: Kinetic and thermodynamic studies. Ind. Crop. Prod..

[14] Wang T., Pan X., Ben W., Wang J., Hou P., Qiang Z. (2017). Adsorptive removal of antibiotics from water using magnetic ion exchange resin. J. Environ. Sci..

[15] Kolpak A.M., Grossman J.C. (2011). Azobenzene–functionalized carbon nanotubes as high energy density solar thermal fuels. Nano Lett..

[16] Wu Y., Zeng Y., Li L., Monfort O., Dong W. (2025). Green Fenton–like degradation of sulfamethoxazole using chlorogenic acid under neutral conditions: A mechanistic and environmental risk perspective. Environ. Chem. Ecotox..

[17] Gunten U.V. (2003). Ozonation of drinking water: Part I. oxidation kinetics and product formation. Water Res..

[18] Dodd M.C., Huang C. (2004). Transformation of the antibacterial agent sulfamethoxazole in reactions with chlorine: Kinetics, mechanisms, and pathways. Environ. Sci. Technol..

[19] Gahrouei A.E., Vakili S., Zandifar S., Pourebrahimi S. (2024). From wastewater to clean water: Recent advances on the removal of metronidazole, ciprofloxacin, and sulfamethoxazole antibiotics from water through adsorption and advanced oxidation processes (AOPs). Environ. Res..

[20] Fatta–Kassinos D., Vasquez M.I., KÜmmerer K. (2011). Transformation products of pharmaceuticals in surface waters and wastewater formed during photolysis and advanced oxidation processes—Degradation, elucidation of byproducts and assessment of their biological potency. Chemosphere.

[21] Wu W., Zhang L., Li Z., Wang C., Yu C., Wang Q. (2021). Research progress of advanced oxidation technology in degradation of antibiotics and removal of antibiotic resistance. Chem. Ind. Eng. Prog..

[22] Wang S., Wang J. (2018). Activation of persulfate (PS) and peroxymonosulfate (PMS) and application for the degradation of emerging contaminants. Chem. Eng. J..

[23] Hu P., Long M. (2016). Cobalt–catalyzed sulfate radical–based advanced oxidation: A review on heterogeneous catalysts and applications. Appl. Catal. B Environ. Energy.

[24] Anipsitakis G.P., Dionysiou D.D. (2004). Radical Generation by the Interaction of Transition Metals with Common Oxidants. Environ. Sci. Technol..

[25] Wang Y., Indrawirawan S., Duan X., Sun H., Ang H.M., Tadé M.O., Wang S. (2015). New insights into heterogeneous generation and evolution processes of sulfate radicals for phenol degradation over one–dimensional α–MnO_2_ nanostructures. Chem. Eng. J..

[26] Virkutyte J., Varma R.S. (2014). Eco–Friendly Magnetic Iron Oxide–Pillared Montmorillonite for Advanced Catalytic Degradation of Dichlorophenol. ACS Sustain. Chem. Eng..

[27] Karthikeyan S., Boopathy R., Sekaran G. (2015). In situ generation of hydroxyl radical by cobalt oxide supported porous carbon enhance removal of refractory organics in tannery dyeing wastewater. J. Colloid Interface Sci..

[28] Fang G., Liu C., Gao J., Dionysiou D.D., Zhou D. (2015). Manipulation of Persistent Free Radicals in Biochar to Activate Persulfate for Contaminant Degradation. Environ. Sci. Technol..

[29] Chen X., Oh W., Lim T. (2018). Graphene–and CNTs–based carbocatalysts in persulfates activation: Material design and catalytic mechanisms. Chem. Eng. J..

[30] Nie C., Wang J., Cai B., Lai B., Wang S., Ao Z. (2024). Multifunctional roles of MoS_2_ in persulfate–based advanced oxidation processes for eliminating aqueous organic pollutants: A review. Appl. Catal. B Environ. Energy.

[31] Brigante M., Pecini E., Avena M. (2016). Magnetic mesoporous silica for water remediation: Synthesis, characterization and application as adsorbent of molecules and ions of environmental concern. Micropor. Mesopor. Mat..

[32] Qu X., Fu H., Mao J., Ran Y., Zhang D., Zhu D. (2016). Chemical and structural properties of dissolved black carbon released from biochars. Carbon.

[33] Fan X., Wu Y., Sun Y., Tu R., Ren Z., Liang K., Jiang E., Ren Y., Xu X. (2022). Functional groups anchoring–induced Ni/MoOx–Ov interface on rice husk char for hydrodeoxygenation of bio–guaiacol to BTX at ambient–pressure. Renew. Energy.

[34] Lyle E.S., Mcallister C., Dahn D.C., Bissessur R. (2020). Exfoliated MoS_2_–Polyaniline Nanocomposites: Synthesis and Characterization. J. Inorg. Organomet. P.

[35] Han X., Gerke C.S., Banerjee S., Zubair M., Jiang J., Bedford N.M., Miller E.M., Thoi V.S. (2020). Strategic Design of MoO_2_ Nanoparticles Supported by Carbon Nanowires for Enhanced Electrocatalytic Nitrogen Reduction. ACS Energy Lett..

[36] Li N., Li R., Duan X., Yan B., Liu W., Cheng Z., Chen G., Hou L., Wang S. (2021). Correlation of active sites to generated reactive species and degradation routes of organics in peroxymonosulfate activation by Co–loaded carbon. Environ. Sci. Technol..

[37] Chen Y., Wu Y., Liu C., Guo L., Nie L., Chen Y., Qiu T. (2018). Low–temperature conversion of ammonia to nitrogen in water with ozone over composite metal oxide catalyst. J. Environ. Sci..

[38] Lu H., Feng W., Li Q. (2022). Degradation Efficiency Analysis of Sulfadiazine in Water by Ozone/Persulfate Advanced Oxidation Process. Water.

[39] Xiang L., Xie Z., Guo H., Song J., Li D., Wang Y., Pan S., Lin S., Li Z., Han J. (2021). Efficient removal of emerging contaminant sulfamethoxazole in water by ozone coupled with calcium peroxide: Mechanism and toxicity assessment. Chemosphere.

[40] Cao J., Jing Y., Du Z., Chu W., Li J., Cen W. (2021). WC/BiOCl binary composite photocatalyst for accelerating interfacial charge separation and sulfamethoxazole degradation. Appl. Surf. Sci..

[41] Huang Y., Yang J. (2021). Degradation of sulfamethoxazole by the heterogeneous Fenton–like reaction between gallic acid and ferrihydrite. Ecotoxicol. Environ. Saf..

[42] Yu S., Gao Y., Khan R., Liang P., Zhang X., Huang X. (2020). Electrospun PAN–based graphene/SnO_2_ carbon nanofibers as anodic electrocatalysis microfiltration membrane for sulfamethoxazole degradation. J. Membr. Sci..

[43] Xiang L., Xie Z., Guo H., Song J., Li D., Wang Y., Pan S., Lin S., Li Z., Han J. (2022). Tunable active sites on biogas digestate derived biochar for sulfanilamide degradation by peroxymonosulfate activation. J. Hazard. Mater..

[44] Wang Y., Zhou C., Wu J., Niu J. (2020). Insights into the electrochemical degradation of sulfamethoxazole and its metabolite by Ti/SnO_2_–Sb/Er–PbO_2_ anode. Chin. Chem. Lett..

